# The lipedema common case report form as a research tool: standardizing lipedema data collection

**DOI:** 10.3389/fgwh.2026.1833913

**Published:** 2026-06-24

**Authors:** Stephanie Galia, Rachelle Crescenzi, Philipp Kruppa, Jonathan Kartt, Jesse C. Cochrane, Courtney Mascio, Laura Harmacek, Stacey Heil, Elena Samouhos, Stephanie Peterson, Steven M. Dean, Thomas F. Wright, Vincenza Cifarelli, Timothy P. Padera, Joseph M. Rutkowski, Katherine S. Eddens, Celia Egan, Sarah Whitehead, Karen Louise Herbst, Bruce A. Bunnell, Carrie Shawber, Tim Hucho, Leslie Hinyard, Sara Al-Ghadban, Allison O. Scott, Pia Ostergaard, Alan Pittman, Pamela A. Nono Nankam, Philipp E. Scherer, Isabel Forner-Cordero, Robert J. Damstra, Ad Hendrickx, Ashok Srinivasan

**Affiliations:** 1 Lipedema Foundation, New York, NY, United States; 2 Department of Radiology and Medical Imaging, University of Virginia, Charlottesville, VA, United States; 3 Department of Plastic, Aesthetic and Reconstructive Microsurgery/Hand Surgery, Hospital Ernst von Bergmann, Potsdam, Germany; 4 Department of Plastic, Reconstructive and Aesthetic Surgery – Hand Surgery and Burn Center, University Hospital Schleswig-Holstein, Lübeck, Germany; 5 Wexner Medical Center, The Ohio State University, Columbus, OH, United States; 6 Lipedema Surgical Solutions, O’Fallen, MO, United States; 7 Health Outcomes Accelerator Group, LLC, St. Louis, MO, United States; 8 Department of Radiation Oncology, Massachusetts General Hospital and Harvard Medical School, Boston, MA, United States; 9 Division of Lymphatic Biology, Department of Medical Physiology, Texas A&M University College of Medicine, Bryan, TX, United States; 10 Department of Epidemiology and Biostatistics, School of Public Health, Indiana University Bloomington, Bloomington, IN, United States; 11 True Women’s Health, Grand Rapids, MI, United States; 12 Lumina Vascular Health & Laser Clinic, Seattle, WA, United States; 13 The Roxbury Institute, Beverly Hills/Tucson, CA/AZ, United States; 14 Department of Biomedical and Translational Sciences, Carle Illinois College of Medicine, University of Illinois Urbana-Champaign, Champaign, IL, United States; 15 Departments of Obstetrics and Gynecology and Surgery at Columbia University Irving Medical Center, New York, NY, United States; 16 Translational Pain Research, Department of Anesthesiology and Intensive Care Medicine, Medical Faculty and University Hospital of Cologne, University of Cologne, Cologne, Germany; 17 Department of Health and Clinical Outcomes Research, Saint Louis University School of Medicine, St. Louis, MO, United States; 18 Department of Plastic Surgery, Maxillofacial & Oral Health, University of Virginia, Charlottesville, VA, United States; 19 Cardiovascular & Genomics Research Institute, City St George's University of London, London, United Kingdom; 20 Helmholtz Institute for Metabolic, Obesity and Vascular Research (HI-MAG) of the Helmholtz Zentrum München at the University of Leipzig and University Hospital Leipzig, Leipzig, Germany; 21 Touchstone Diabetes Center, University of Texas Southwestern Medical Center, Dallas, TX, United States; 22 Hospital Universitari i Politècnic la Fe, University of Valencia, Valencia, Spain; 23 Center of Expertise for Lymphovascular Medicine, Nij Smellinghe Hospital, Drachten, Netherlands

**Keywords:** Biobank, biorepository, CCRF, common case report form, data collection, lipedema, lipedema diagnosis, Lipedema Foundation

## Abstract

Lipedema is a chronic adipose tissue condition that primarily affects women. Despite increasing recognition of lipedema, the condition remains poorly understood and lacks standardized diagnostic criteria or confirmatory tests. Variability in definitions and measurement across clinical and research settings impedes comparability across studies, constraining the evidence base needed to support future advances in clinical practice and patient care. To address challenges associated with inconsistent definitions and data collection, the Lipedema Foundation (LF) partnered with clinicians, researchers, and biostatisticians to develop a Lipedema Common Case Report Form (CCRF). The CCRF was designed to be a research data harmonization tool and is not intended to define diagnostic standards or guide clinical treatment decisions. Its development involved review of published lipedema clinical guidelines and collaborative work to define data elements and attributes for inclusion. When they existed, validated or standardized measures were incorporated directly. When no suitable standardized measures were available, an iterative and collaborative process was used to develop lipedema-specific Common Data Elements (CDEs). The initial version of the CCRF was piloted in participants with and without lipedema, and updates based on participant and clinician feedback were incorporated into the CCRF. A biostatistical review evaluated data completeness, quality, and structure, leading to additional refinements. The final Version 1 instrument consists of 682 CDEs organized into four classifications: (1) Core, (2) Supplemental Highly Recommended, (3) Supplemental, and (4) Exploratory. The current version is prepared for dissemination in the field. By disseminating the CCRF broadly and encouraging adoption in all lipedema research beginning in 2026, including all newly initiated LF-funded projects, LF intends to evaluate its use with grantees and iterate systematically to achieve consistent and comparable data collection. The CCRF provides a structured framework for harmonized data collection that may facilitate comparability across studies and support future development of standardized diagnostic and research methodologies.

## 1 Introduction

Lipedema is a chronic adipose tissue condition primarily affecting women, characterized by bilateral, symmetric, and disproportionate subcutaneous fat deposition in the lower extremities and, in some cases, the arms and lower trunk (hips, buttocks, and abdomen). The feet and hands are typically spared, sometimes causing the appearance of an ankle or wrist “cuff” (1–7). Reported prevalence estimates vary widely depending on diagnostic criteria and study design, with estimates ranging from approximately 1% to 10% in selected populations. This variability highlights the lack of standardized diagnostic approaches (8–12). For consistency, lipedema is referred to as a condition throughout this manuscript; however, the terms disease and condition are often used interchangeably in the literature, and some authors prefer the terms disorder or syndrome.

Lipedema is typically categorized into three stages (Stages 1–3) based on morphological characteristics, including changes in skin texture, nodularity, fibrosis, and abnormal adipose deposition. Staging is most commonly based on findings in the lower extremities. Although this framework is widely used and supported by most authoritative sources, staging definitions vary in detail across guidelines, and emerging perspectives have begun to question or expand upon traditional stage-based classification, because their correlation with clinical symptoms can be limited (1, 3, 13–22). Lipedema is frequently misdiagnosed or conflated with obesity and lymphedema, contributing to delays in diagnosis and inconsistent clinical management (1–6).

Individuals with lipedema may experience fatigue, pain, tenderness, sensory changes, swelling, easy bruising, and hypermobility. The intensity of pain may range from none to severe, with pain being constant, intermittent, or only occurring when the affected areas are palpated (23, 24). Pain and/or discomfort may be accompanied by an unusual nodular and/or fibrotic texture within the subcutaneous fat (1–6). Survey-based studies indicate that many patients consult multiple healthcare providers before receiving a diagnosis, often after prolonged diagnostic delays. These delays are associated with inconsistent clinical management and a substantial disease burden, including daily pain, mobility loss, reduced quality of life, body shame, disrupted social and intimate relationships, and significant psychological distress (18, 24–28).

The causes of lipedema are not well understood, but patient reports suggest that onset and condition worsening most frequently occur during puberty and other periods of hormonal change, such as pregnancy and menopause (18, 26, 27). Ongoing research is examining the roles of hormones, genetics, metabolism, and lymphatic involvement in etiology and pathogenesis.

**Figure 1:**
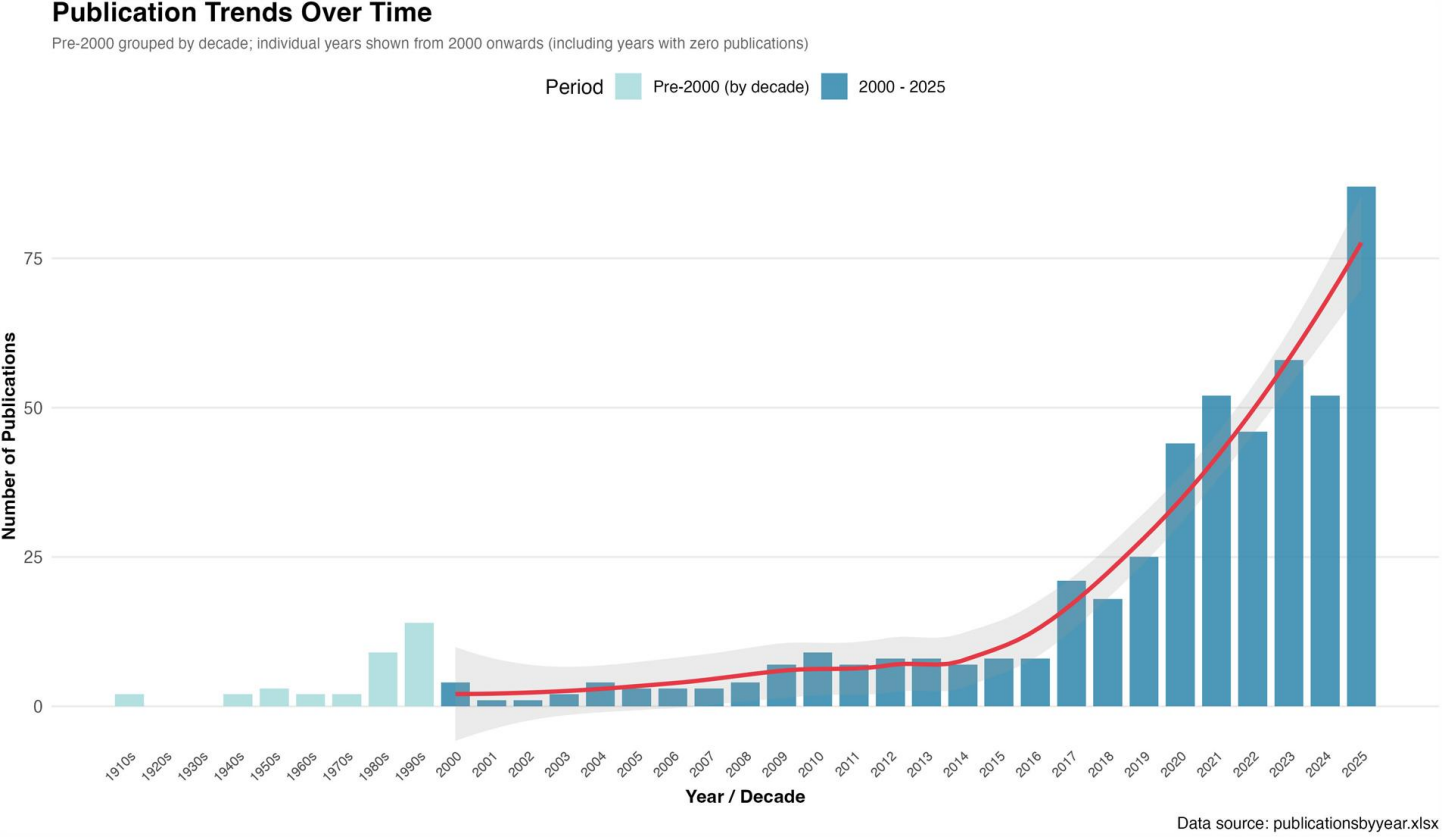
Original research publication growth in lipedema over time. Annual number of PubMed-indexed primary data publications related to lipedema from 1910 through 2025. Although early literature is very limited, publication volume has increased substantially in recent years, with approximately 56% of all indexed publications appearing within the last five years (2021–2025), reflecting rapid growth in a field that remains in an early stage of development.

The volume of primary research on lipedema has increased substantially in recent years (Figure 1); however, the total number of indexed publications remains limited compared with other chronic adipose tissue or metabolic disorders. Estimates from PubMed searches indicate a marked increase in publications since 2020, reflecting growing scientific interest; however, this increasing research activity has occurred in the absence of consistent diagnostic criteria and validated tools, limiting reproducibility and slowing clinical progress. Research efforts are further hindered by inconsistent data collection practices, with investigators using different questionnaires, measures, and data elements. To date, no data collection instrument exists to support capture of diagnostic, clinical, and patient-reported data in lipedema research.

Lipedema is characterized not only by distinct morphological features of adipose tissue distribution but also by a range of physical symptoms, participation restrictions, and functional limitations, including pain, swelling, and impaired mobility. These multidimensional manifestations align with the World Health Organization's International Classification of Functioning, Disability and Health (ICF) framework, which integrates biological, functional, and psychosocial domains (29). As lipedema impacts more than physical health, it is critical that standardized data collection approaches capture not only biological domains, but also functional and psychosocial domains. This more expansive approach to data collection is infrequently deployed in the field of lipedema research.

Variability in definitions and measurement complicates comparisons between studies, impedes meta-analyses, and increases the cost and complexity of statistical analyses. The absence of standards inhibits the establishment of uniform diagnostic criteria and the rigorous development and evaluation of therapeutic interventions, constraining the accumulation of evidence needed to inform patient care. As a result, fundamental questions remain unresolved, including how to characterize condition heterogeneity, how to define and identify clinically relevant subgroups, how to stage severity, how to assess pain and functional impairment, and how the condition progresses over time. Without standardized data and consistent diagnostic approaches, healthcare payers and policymakers often characterize the existing evidence base as low quality or inconclusive, creating additional barriers for patients with lipedema seeking appropriate treatment.

To address these challenges, the LF partnered with global researchers and clinicians to develop a CCRF as a framework for harmonized data capture in lipedema research. The CCRF was developed through an iterative, collaborative process to define CDEs, incorporating validated measures when available and developing lipedema-specific elements when necessary. Here, we describe the development, piloting, and classification framework of the CCRF and present Version 1 of the instrument, a tool designed to support standardized data collection rather than establish or validate clinical diagnostic criteria.

## 2 Materials and equipment

The development and implementation of the CCRF required referencing of established CDE repositories, validated instrument libraries, and published surveys; the use of digital platforms; and research infrastructure to support standardized data definitions, instrument building, and deployment.

### 2.1 CDE repositories and published instruments

Established CDE repositories, validated instrument libraries, research priority documents, and published surveys were referenced to inform standardized question selection, variable definitions, and response formats. Notable references included the LF Research Roadmap, LF Patient Registry, National Institutes of Health (NIH) CDE Repository, and the United States Core Data for Interoperability (USCDI). (See more details about process and an inclusive list of references in Section 3.3 Preparatory Research and Source Standards.)

### 2.2 Software and digital platforms

The CCRF instrument was developed and deployed using REDCap Cloud. Stakeholders collaborated via Google Workspace and Zoom. Reference management and literature organization were supported through Zotero, a digital library and citation management system. Statistical review and data quality assessment were conducted using established analytic software, including R, RStudio, SAS, and Python.

### 2.3 Infrastructure and analytical tools

Pilot implementation utilized the LF Biobank infrastructure, including temporary clinical examination space created within a hotel conference setting (ballroom) using pipe-and-drape partitions. This set-up supported private, standardized clinical assessments, as well as participant completion of self-administered survey components in the same controlled environment, with appropriate data collection support. Clinical measurements were obtained using a calibrated digital scale, a non-contact infrared thermometer, and standardized tape measures.

### 2.4 Generative AI assistance

Generative AI tools were used to support manuscript preparation and figure development. ChatGPT (GPT-5–class model, 2026; OpenAI, https://openai.com) assisted with manuscript writing and editing, while Claude Sonnet 4.6 (Anthropic, https://anthropic.com) supported the development of the flowchart. All AI-assisted content was reviewed and verified by the authors for factual accuracy and plagiarism, and the authors take full responsibility for the integrity of the final content.

## 3 Methods

### 3.1 Governance and oversight

CCRF development was led by LF, with input from clinical, research, and biostatistics experts. Oversight included structured internal reviews, and external consultation with international stakeholders and the LF Scientific Advisory Committee (SAC). This structure was established to ensure scientific rigor, clinical relevance, and transparency throughout the development process. The LF Biobank Live Research event represented the first biological sample and data collection activities of the LF Biobank.

### 3.2 Stakeholder inputs

Broad stakeholder engagement was central to CCRF development. External contributors and reviewers included clinicians, surgeons, and clinician-researchers, as well as scientists with expertise in pain, imaging, adipose biology, lymphatic biology, and areas of basic science relevant to lipedema.

Participation in CCRF development activities was voluntary. Clinicians, researchers, and other stakeholders who contributed to review and CCRF refinement did so without financial compensation related to this project. Members of the LF SAC may receive honoraria for service to LF; however, no honoraria were provided in connection with CCRF development activities. Research participants who completed the CCRF as part of the LF Biobank Live Research event received a modest financial incentive for study participation, but no incentives were provided specifically for feedback on the instrument.

### 3.3 Preparatory research and source standards

Development of the CCRF required systematic identification, review, and synthesis of existing data standards, published instruments, and condition-specific literature to draft and refine CDEs appropriate for lipedema research. Preparatory work began with compilation of candidate variables informed by LF's Lipedema Research Roadmap, which consolidated input from more than 60 global stakeholders (30). Existing standardized sources were reviewed, including the U.S. Core Data for Interoperability (USCDI) (31), the NIH CDE Repository (32), the International Classification of Functioning, Disability and Health (29), Systematized Nomenclature of Medicine (SNOMED) (33), U.S. Office of Management and Budget race and ethnicity standards (34), and the NIH Classifications of Data Elements for a Specific Disease (35). Additional reference materials included Cleveland Clinic test-specific sites (36, 37), SF-RAND Quality of Life Surveys (38), the LF Patient Registry Survey (39), and published lipedema guidelines, standards, and foundational publications informing diagnostic and staging approaches (Table 1). To support standardized assessment of symptom severity, the CCRF incorporated a slightly modified version of the Lipedema Symptom Scale, originally developed by Rapprich et al. (40) and subsequently applied in lipedema research, including by Verde et al. (41). In alignment with the World Health Organization's bio-psycho-social model, select elements were informed by the International Classification of Functioning, Disability and Health (ICF) (29) to ensure inclusion of functional and psychosocial domains alongside clinical measures. Validated measures were incorporated whenever available and appropriate. Metric units were used wherever applicable to increase international usability and support comparability across global research settings.

**Table 1:** Key publications informing lipedema diagnostic and staging criteria.

Author(s)	Year	Publication type	Use in CCRF development
Allen and Hines	1940	Original clinical description	Diagnosis
Wold et al.	1951	Clinical description and observational report	Diagnosis
Strößenreuther and Baumeister	2001	Clinical monograph	Diagnosis; Staging
Schmeller and Meier-Vollrath	2008	Textbook chapter	Diagnosis; Staging
Schmeller and Meier-Vollrath	2009	Clinical textbook chapter	Diagnosis; Staging
Fife et al.	2010	Clinical review	Diagnosis; Staging
Herbst	2012	Review of rare adipose disorders	Diagnosis
Damstra et al.	2014	National clinical guideline (Netherlands)	Diagnosis; Staging
Herbst et al.	2015	Observational study	Staging
Buck and Herbst	2016	Narrative review	Diagnosis
Reich-Schupke et al.	2017	National clinical guidelines (Germany, Version 1)	Diagnosis; Staging
Wounds UK	2017	National consensus document (UK)	Diagnosis; Staging
Alcolea et al.	2018	National consensus document (Spain)	Diagnosis; Staging
Bertsch and Erbacher	2020	Review article	Diagnosis; Staging
Herbst et al.	2021	National clinical guidelines (U.S.)	Diagnosis; Staging
Kruppa et al.	2023	Mechanistic/histopathologic study	Staging
Lipedema Foundation	2023	Research roadmap	Staging
Faerber et al.	2024	National clinical guidelines (Germany, Version 2)	Diagnosis; Staging
German Society for Phlebology and Lymphology	2024	S2 clinical guideline	Diagnosis; Staging
Keith et al.	2024	Research case definition framework	Diagnosis; Staging
Cifarelli	2025	Review article	Diagnosis
Funcke et al.	2025	Mechanistic review	Diagnosis
Rabiee	2025	Review article	Diagnosis

### 3.4 Development of common data elements

The CCRF is a structured instrument composed of standardized questions that capture individual CDEs. Aspects of CDEs that required standardization include variable name, digital format (characters and units), numeric range, text entry length, and variable scales.

CDEs were developed through an iterative and collaborative process involving clinicians, researchers, and data analysts. Where they existed, standardized or validated measures were incorporated into the CCRF directly. When no suitable standardized measure was available, lipedema-specific CDEs were drafted, edited, and finalized through an extensive review process to capture clinically relevant diagnostic, descriptive, and patient-reported information.

Item wording, decisions regarding item inclusion in the CCRF, response options, and branching logic were refined through repeated review cycles informed by stakeholder feedback and preliminary pilot implementation. All items subsequently underwent item-level statistical analysis to support further refinement of the instrument. (See Section 3.9 Statistical Review and Refinement).

### 3.5 CCRF structure and intended respondents

The CCRF was designed as a comprehensive instrument composed of distinct participant-completed and clinician-completed sections. Participant-facing sections were intended to be self-administered and to capture demographic information, medical and family history, medication use (e.g., hormone, weight loss/diabetes), comorbidities, and lipedema-specific history, including symptom burden, onset, progression, and reported triggers.

Clinician-facing sections were structured to capture measurement-based data, diagnostic characterization, and clinical findings commonly associated with lipedema. Measurement components (e.g., height, weight, temperature) were designed to be completed by trained research or medical personnel when appropriate, while diagnostic assessments, staging elements, palpable findings, and other clinical observations intended for research classification required input from a diagnosing clinician.

### 3.6 Stakeholder review process

The CCRF underwent a multi-phase review and refinement process. LF staff, as well as data management partners, and experienced clinicians and researchers, conducted initial reviews to refine item structure, logic, and content. This process was followed by piloting of the CCRF during the LF Biobank Live Research event, during which feedback was collected from research participants and diagnosing clinicians who completed the instrument in practice; this feedback informed further revisions to CCRF wording, flow, and usability. Subsequently, CDEs were categorized through a collaborative classification process involving LF staff, the LF SAC, and key international researchers, with the goal of broad acceptance as the minimum standard for final inclusion and prioritization of Core elements.

### 3.7 User acceptance testing and piloting

User acceptance testing (UAT) was conducted by Lipedema Foundation staff, including staff with lipedema, to evaluate usability prior to implementation of the CCRF in the LF Biobank and again following biostatistical review.

### 3.8 Pilot at live research event

The CCRF was field tested during the first LF Biobank Live Research event. This in-person research event involved human research participants and was held during the Fat Disorders Resource Society 2025 Annual Conference. Research procedures were conducted as approved by an Institutional Review Board (Northstar #NB400218).

### 3.9 Statistical review and refinement

Statistical review was conducted to evaluate the structure, completeness, and analytic readiness of the CCRF and to inform its refinement. Three biostatisticians and one epidemiologist-biostatistician reviewed CCRF data generated during the pilot LF Biobank Live Research event. Analyses included assessment of item completeness and patterns of missingness, evaluation of response distributions, review of branching logic and internal consistency, and identification of potential data quality issues such as outliers or inconsistent responses.

CDE-level analyses were performed to examine response behavior and to assess user-facing features, construct, and structural validity where applicable. When appropriate, responses to individual items were compared with related items within the CCRF or with established external instruments to assess alignment with expected constructs.

For scale-based measures included in the CCRF, analyses included evaluation of internal consistency and reliability, as well as exploratory factor and clustering analyses to assess underlying structure.

### 3.10 Classification of common data elements

Following piloting and statistical review, CDEs were categorized to support use across diverse study designs while enabling flexibility for evolving research needs. Classification was modeled after the NIH CDE classification framework (32) and included four categories: (1) Core, (2) Supplemental Highly Recommended, (3) Supplemental, and (4) Exploratory.

Classification decisions were made through a collaborative review process involving LF staff, LF SAC members, and key international clinicians and researchers. The NIH CDE classification framework served as the initial structural model, with definitions subsequently adapted to reflect the specific needs of the lipedema field. Considerations included clinical relevance, frequency of use across published studies, importance for diagnostic characterization and comparability, participant burden, and feasibility of collection across diverse research and clinical settings (Table 2).

**Table 2:** Classification of common data elements (CDEs) in the CCRF.

CDE classification	Definition	Number of CDEs
Core	Common data elements related to the diagnosis of Lipedema include items essential for identifying, confirming, or differentiating Lipedema, as well as those providing clinically relevant information for diagnosis. Core elements include clinically assessed items such as Lipedema diagnosis for research purposes, Lipedema stage, anthropometric measurements, presence of edema, and palpable findings of nodularity or fibrosis. Participant-reported items that support diagnosis are also included, such as pain or discomfort in affected areas, common comorbidities, current medications and supplements, surgical history, menstrual status, family history, and basic demographics. This classification addresses areas of clinical disagreement and research gaps, facilitating data collection for cross-study and cross-geography comparisons to enhance understanding.	**Total CDE’s: 288** 86 Clinical Form (∼13 min to complete) 202 Participant Form (∼16 min to complete)
Supplemental – highly recommended	**Core +** Data elements that are not required for a basic diagnosis of Lipedema but are strongly recommended for comprehensive characterization of the condition and/or samples. These elements provide important context about subpopulations/subtypes and foundational understanding of disease onset, worsening (progression or exacerbation), severity, or impact and are widely applicable across most Lipedema studies.	**Total CDE’s: 424** 89 Clinical Form (∼13 min to complete) 335 Participant Form (∼19 min to complete)
Supplemental	**Core + Supplemental - Highly Recommended +** Data elements that may add scientific or clinical value but are not critical to diagnosis or characterization in all studies. These are useful in specific research aims or contexts, such as exploring specifics (such as triggers) for disease onset or worsening, treatment outcomes, quality of life, or comorbid conditions.	**Total CDE’s: 618** 89 Clinical Form (∼13 min to complete) 529 Participant Form (∼42 min to complete)
Exploratory	**Core + Supplemental - Highly Recommended + Supplemental +** Data elements that are emerging, experimental, or do not yet have a clear link to Lipedema. These elements may represent novel assessments, biomarkers, or patient-reported outcomes not yet validated with Lipedema populations. Exploratory items can inform hypothesis generation and future standardization but are not yet recommended for routine use.	**Total CDE’s: 682** 120 Clinical Form (∼18 min to complete) 562 Participant Form (∼45 min to complete)

Decisions were reached through broad agreement rather than formal consensus procedures, with the goal of defining a minimum recommended standard for data collection while preserving flexibility for hypothesis-driven and exploratory research about lipedema. Final decisions about classification definitions and the CDEs to be included in each classification were made by LF staff and incorporated all feedback from stakeholders.

### 3.11 Accessibility

**Figure 2:**
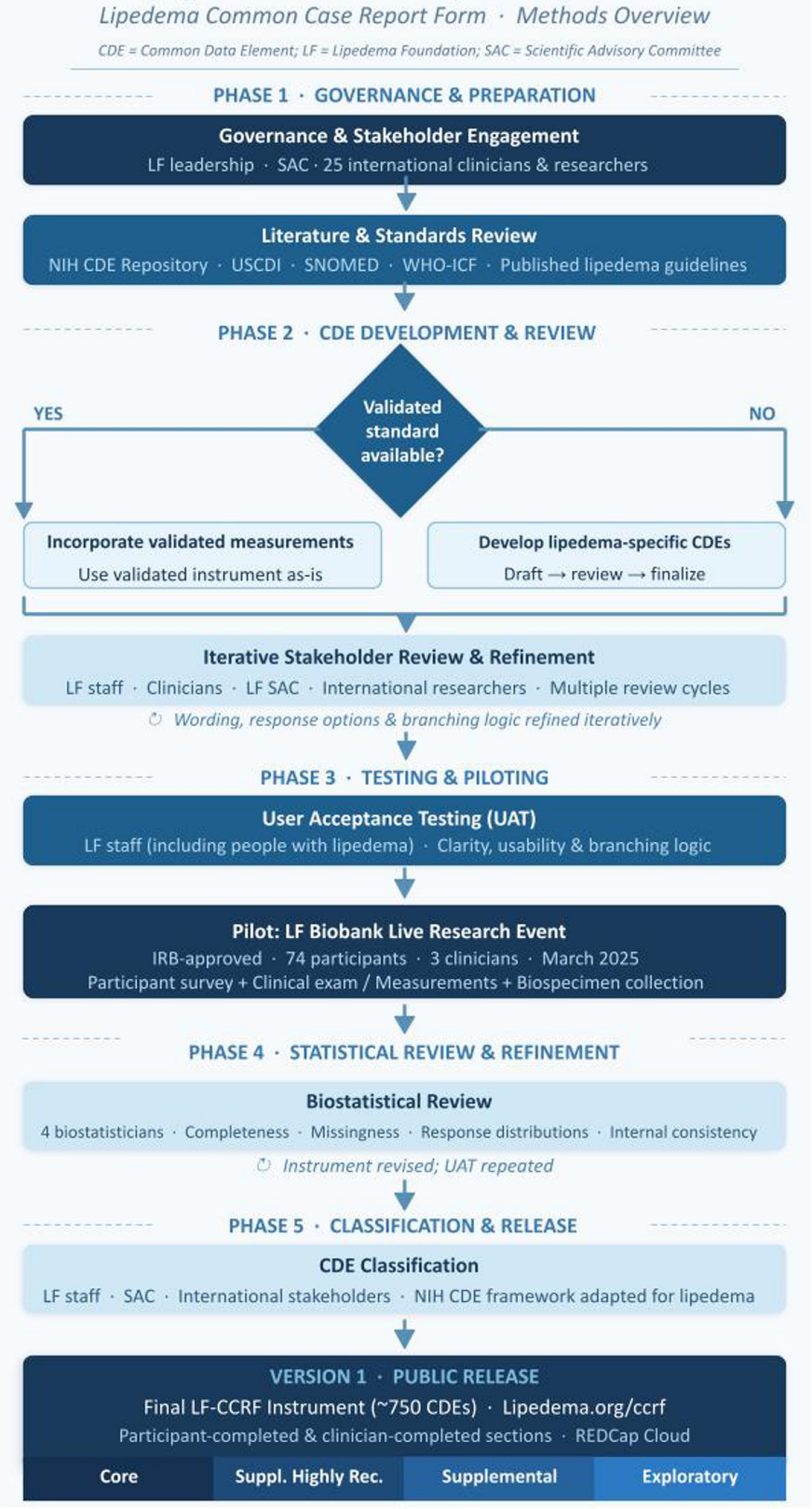
CCRF development process.

Accessibility considerations included incorporating plain-language, patient-facing sections and a streamlined form design to minimize burden for participants, clinicians, and LF staff. At this time, the form is available in Standard American English. In practice, the CCRF was completed digitally in REDCap Cloud, and paper CCRF forms were made available in case of connectivity issues. The overall development process for the CCRF is summarized in Figure 2.

## 4 Results

Results are presented in the order corresponding to the steps of the CCRF development process, including stakeholder participation, pilot implementation, statistical evaluation, and final instrument creation. The primary outputs of this work were (1) development of a comprehensive, standardized CCRF database for lipedema research and (2) establishment of a tiered classification system for CDEs.

### 4.1 Project timeline and development phases

CCRF development spanned approximately 22 months, from initial conceptualization to public release. Early drafting of candidate variables and database construction occurred during months 1–3, followed by internal review at the LF and iterative refinement. Broad external stakeholder review and revisions occurred during months 7–9. Instrument build and UAT occurred during months 10 and 11. Piloting of the CCRF during the LF Biobank Live Research event occurred during month 11. Subsequent review of published diagnostic criteria guidelines, statistical evaluation, and instrument refinement occurred during months 12 and 17. Final updates, re-testing, and classification of CDEs occurred during months 18–20, with updates executed and public release of Version 1 by month 22.

### 4.2 Stakeholder participation

Stakeholder participation included 25 external reviewers with expertise in lipedema care and research, three diagnosing clinicians who completed clinical components during live piloting, three biostatisticians, and one epidemiologist-biostatistician. External reviewers represented a range of disciplines, including clinical care, surgery, pain research, biomedical imaging, adipose biology, lymphatic biology, and related fields.

The diverse stakeholder engagement reflects the multidisciplinary scope of lipedema research and limited availability of standardized measures in the field, with heterogeneity in diagnosis, staging, comorbidity documentation, CDE wording, and element inclusion. The pilot included a trained physician and 74 research participants—66 individuals with lipedema and 8 individuals without lipedema who also provided feedback.

### 4.3 CCRF composition and structure

The final Version 1 CCRF consists of 682 CDEs spanning participant-reported, clinician-reported, and measurement-based domains. This organization was undertaken to balance clinical utility, participant burden, and analytic rigor. This comprehensive instrument is applicable across multiple scientific domains. Following piloting and refinement, CDEs were classified into four categories.

### 4.4 User acceptance testing

User acceptance testing evaluated usability, clarity of item wording and response options, and appropriate branching logic across participant- and clinician-facing sections of the instrument, while also assessing overall functionality, interpretability, and feasibility for implementation in research settings.

### 4.5 Piloting and implementation

Feedback from pilot implementation informed further refinements to item structure, flow, and answerability prior to finalization.

All CDEs were successfully implemented during the pilot phase. Completion rates were high, supporting feasibility of implementation, although additional testing in larger and more diverse cohorts will be required. Review of response patterns suggested that items were interpretable by participants and clinicians, supporting use of the CCRF in future research settings.

### 4.6 Statistical review and item-level findings

Statistical evaluation identified patterns of missingness, response distributions, and item behavior across participant- and clinician-completed (diagnostic and measurement) sections. Item branching logic and response consistency were reviewed, and outliers and unusual distributions were identified for further consideration. These statistical assessments were conducted to support refinement of item wording, response options, guidance, and structure, and were not intended to establish definitive clinical validation of the CCRF or its components.

Additional analyses were conducted for scale-based measures included in the CCRF. The Lipedema Symptom Scale, modified according to Rapprich et al. (40) showed internal consistency across pilot participants. Item-item correlations, Cronbach's alpha (raw and standardized), and Guttman's Lambda 6 were calculated for the 15-item score. Reliability analyses were repeated with each item removed to assess item contribution to scale performance. Exploratory factor analysis and hierarchical cluster analysis were conducted among participants with lipedema to evaluate underlying structure.

### 4.7 Refinements resulting from review and analysis

Findings from stakeholder review, piloting, and statistical evaluation informed targeted refinements to both participant-facing and clinician-facing sections of the CCRF. Updates included clarification of item wording, removal or consolidation of redundant items, adjustments to branching logic, and refinement of instructions for completing CDE elements.

### 4.8 Final instrument release

Following incorporation of stakeholder feedback and statistical findings, the CCRF was finalized and made publicly available through the LF website (lipedema.org/ccrf). Materials include the full CCRF instrument, classification-specific versions, and a complete data dictionary published in comma-separated values (.csv) format to facilitate reuse.

The complete instrument, classification-specific subsets, and accompanying data dictionary are publicly available in standardized formats. Adoption of the CCRF is strongly encouraged across the field, including in newly initiated LF-funded research projects beginning in 2026. Researchers using the CCRF are encouraged to provide structured CCRF datasets as supplemental material alongside publications.

## 5 Discussion

The overall goal of developing a CCRF for lipedema is to support methodological consistency in the field of lipedema. This work presents the first collaboratively developed, stakeholder-informed CCRF for lipedema research. Although prior publications have advanced clinical guidelines, standards of care, and research recommendations, no standardized, publicly available CRF has previously been published in the field of lipedema. In developing the CCRF, LF drew on successful CDE and CRF frameworks established in other disease areas, and adapted these approaches to the unique clinical, biological, and patient-reported features of lipedema. The CCRF integrates standardized data elements where available with lipedema-specific CDEs developed through expert collaboration. The CCRF offers a solution to longstanding methodological fragmentation in the field and provides a standardized framework for data collection across studies and research settings.

A major strength of this effort is the collaborative, iterative, inclusive development process, which engaged clinicians, researchers, biostatisticians, and individuals with lipedema. LF played an active expert role in guiding the development and prioritization of CDEs, drawing on its extensive experience in lipedema research coordination, data infrastructure development, and translational science. Multiple rounds of review, live piloting, user acceptance testing, and statistical evaluation supported both practical usability and analytic readiness of the instrument. The tiered classification of CDEs into (1) Core, (2) Supplemental Highly Recommended, (3) Supplemental, and (4) Exploratory categories enables flexible implementation while promoting standardization and comparability across studies.

This methodological study has several limitations. Classification decisions were based on stakeholder input and discussion rather than a formal consensus methodology; therefore, the CCRF should be considered a Version 1 framework subject to ongoing refinement.

The pilot data collected at the LF Biobank Live Research event were modest in size. All pilot participants reported female sex and identified as women. Participants represented a geographically dispersed cohort, with the largest proportion residing in the U.S. state of Georgia and additional representation across multiple U.S. states and three international locations. Despite this geographic spread, most participants identified as White, with smaller numbers identifying as Middle Eastern/North African, Hispanic, Black, or Native American. Importantly, this limitation reflects broader gaps in the current understanding of lipedema epidemiology, including its geographic distribution, prevalence across populations, and expression among individuals of different genetic ancestries. The observed demographic patterns therefore highlight both the practical constraints of recruitment in an undercharacterized condition area and the need for expanded outreach to improve representation in future CCRF implementations.

The importance of consistent and comprehensive collection of diagnostic measures, along with standardized criteria for research use, was highlighted by discrepancies observed in certain clinical measures across diagnosing clinicians. This variability may reflect differences in clinical judgment and/or data collection procedures.

Further, some domains (particularly functional, social, and emotional measures) remain less extensive than biological and clinical components of the CCRF. These limitations underscore the need for continued iteration as the evidence base and utilization of the tool grows.

Despite these limitations, the CCRF may facilitate standardized data capture and improve comparability across lipedema studies. Consistent use of a common data collection framework will reduce barriers to cross-study comparison, support meta-analyses, and enable evaluation of hypotheses related to condition heterogeneity, staging, progression, and patient-reported outcomes, while establishing the CCRF as a shared community resource for lipedema research. Given the ongoing evolution of diagnostic criteria across countries and published guidelines, use of a common CRF will facilitate comparability even when studies operate under differing diagnostic frameworks. The tiered structure further supports stability over time, as Core CDEs are intended to remain consistent and are not expected to change substantially without structured review and broad consensus. Meanwhile, flexibility is enabled through Supplemental and Exploratory elements as the evidence base evolves. Over time, the accumulation of standardized research data generated through the CCRF may also inform the development of clinical assessment tools and support more consistent diagnostic practices as evidence and consensus evolve.

Future work will include expanding international consensus through collaboration with national and international lipedema organizations. As additional data are collected and LF grantees utilize the CCRF, repeat statistical analyses will be conducted to further validate and refine the CDEs, including expansion of control cohorts that provide reference data to improve statistical power and comparison across sites to ensure inter-rater reliability. Additional priorities include refining diagnostic criteria using accumulated participant data, and evaluating CCRF adoption and performance across multiple research sites. LF plans to assess and undertake updates to the CCRF at three year intervals.

## 6 Conclusion

Development of the Lipedema CCRF represents an essential advancement in lipedema research infrastructure. Incorporating international collaboration and broad stakeholder engagement, the CCRF provides a unified, publicly available framework to collect data across lipedema research studies. Organized into the four CDE classifications, the CCRF balances flexibility with standardization to support research across a wide range of designs and settings. By enabling consistent and transparent data collection, the CCRF is intended to facilitate cross-study comparisons and meta-analyses, support methodologically rigorous research, and serve as a shared community resource for evidence generation. As adoption expands in 2026 and beyond, this common framework is expected to strengthen the evidence base upon which future advances in treatment, policy, and patient care may be built.

## Data Availability

The original contributions presented in the study are can be found at lipedema.org/ccrf, further inquiries can be directed to the corresponding author. The Lipedema CCRF, including the full instrument, classification-specific subsets (Core, Supplemental Highly Recommended, Supplemental, and Exploratory), and the accompanying data dictionary in .csv format, is publicly available at lipedema.org/ccrf. Statistical analysis available upon request. Researchers utilizing the CCRF are encouraged to include structured CCRF datasets as supplemental materials with their publications.Users of the CCRF or any of its component CDEs are requested to cite this publication. The CCRF is versioned, and users are encouraged to reference the specific version used in their analyses.
